# Auxin Response Factors promote organogenesis by chromatin-mediated repression of the pluripotency gene *SHOOTMERISTEMLESS*

**DOI:** 10.1038/s41467-019-08861-3

**Published:** 2019-02-21

**Authors:** Yuhee Chung, Yang Zhu, Miin-Feng Wu, Sara Simonini, Andre Kuhn, Alma Armenta-Medina, Run Jin, Lars Østergaard, C. Stewart Gillmor, Doris Wagner

**Affiliations:** 1 Department of Biology, University of Pennsylvania, Philadelphia, PA 19104 USA; 2 Crop Genetics Dept, John Innes Centre, Norwich Research Park, NR4 7UH Norwich, Norfolk UK; 3 Laboratorio Nacional de Genómica para la Biodiversidad (Langebio), Unidad de Genómica Avanzada, Centro de Investigación y de Estudios Avanzados del Instituto Politécnico Nacional (CINVESTAV-IPN), Irapuato C.P., 36824 Guanajuato Mexico; 4 Present Address: Bayer Crop Science, St. Louis, MO 63146 USA; 5 Present Address: Department of Plant and Microbial Biology, University of Zurich, Zollikerstrasse 107, CH-8008 Zurich, Switzerland

## Abstract

Specification of new organs from transit amplifying cells is critical for higher eukaryote development. In plants, a central stem cell pool maintained by the pluripotency factor SHOOTMERISTEMLESS (STM), is surrounded by transit amplifying cells competent to respond to auxin hormone maxima by giving rise to new organs. Auxin triggers flower initiation through Auxin Response Factor (ARF) MONOPTEROS (MP) and recruitment of chromatin remodelers to activate genes promoting floral fate. The contribution of gene repression to reproductive primordium initiation is poorly understood. Here we show that downregulation of the *STM* pluripotency gene promotes initiation of flowers and uncover the mechanism for *STM* silencing. The ARFs ETTIN (ETT) and ARF4 promote organogenesis at the reproductive shoot apex in parallel with MP via histone-deacetylation mediated transcriptional silencing of *STM*. ETT and ARF4 directly repress *STM*, while MP acts indirectly, through its target *FILAMENTOUS FLOWER* (*FIL*). Our data suggest that – as in animals- downregulation of the pluripotency program is important for organogenesis in plants.

## Abstract (web summary)

The pluripotency factor SHOOTMERISTEMLESS (STM) maintains stem cell identity in the centre of the shoot apical meristem. Here Chung et al. show that Auxin Response Factors work in concert with the YABBY transcription factor FIL to silence *STM* via chromatin modifications and allow initiation of primordia that lead to formation of flowers.

## Introduction

Plants give rise to new organs continuously throughout their life. During reproduction, primordia that give rise to flowers and cryptic bracts (henceforth termed reproductive primordia), arise from a population of transit amplifying cells at the flanks of the inflorescence shoot apex^1,2^. Flowers are plant organs important for reproductive success and yield^3^. Perception of a local auxin maximum triggers transcriptional responses that enable specification of reproductive primordium founder cells^4,5^. When auxin levels are low, small nuclear Aux/IAA proteins bind to MONOPTEROS/Auxin Response Factor 5 (MP; ARF5) and recruit co-repressor complexes to prevent activation of auxin responsive genes^6,7^. Increased auxin levels lead to ubiquitin-mediated degradation of Aux/IAA proteins^8^, releasing the co-repressors and allowing MP to recruit the SWI/SNF family chromatin remodelers BRAHMA and SPLAYED (SYD)^9^. In this fashion, MP directly upregulates genes important for flower development, such as those encoding the transcription factors FILAMENTOUS FLOWER (FIL) and LEAFY (LFY)^9,10^. MP also directly modulates expression of genes that control hormone accumulation and response^11–13^.

Auxin Response Factors are divided into three evolutionarily conserved classes, A, B and C^14^. Class A ARFs are classified as transcriptional activators, while class B and C ARFs are classified as transcriptional repressors^15,16^. MP is a class A ARF with an unstructured glutamine-rich central domain that can interact with SWI/SNF family chromatin remodelers^9^. Besides MP, only two other ARFs are strongly expressed in founder cells of the reproductive primordia: *ETTIN* (*ETT*; also called *ARF3*) and *ARF4*^17^. Both ETT and ARF4 are ‘repressive’ class B ARFs^15,16^. ETT and ARF4 have partly redundant roles in several developmental processes in *Arabidopsis*, including lateral root initiation and leaf polarity^18–20^.

A key pluripotency gene in plants is the class I KNOX homeobox transcription factor *SHOOTMERISTEMLESS* (*STM*)^21^. STM promotes meristematic fate in part by upregulating biosynthesis of the hormone cytokinin, which acts in a positive feedback loop to promote stem cell fate^22,23^. *STM* frequently acts in concert with other class I KNOX genes such as *BREVIPEDICELLUS* (*BP*) and the activity of the class I KNOX proteins is modulated by a different class of homeodomain proteins, the BEL-like proteins (see^24^ for review). *STM* expression is high throughout the shoot apical meristem, but is downregulated at sites of auxin maxima in transit amplifying cells^25,26^. Neither the functional significance nor the mechanism for *STM* downregulation in primordium initials are currently understood.

We show here that *STM* repression promotes initiation of reproductive primordia. We further demonstrate that the class B ARFs ETT and ARF4, which are expressed in incipient reproductive primordia, act in parallel with MP to downregulate *STM* as well as *BP*. We implicate the MP target FIL in direct repression of the class I KNOX genes together with ETT and ARF4. Finally, we reveal that silencing of the pluripotency gene *STM* and *BP* by FIL and ETT/ARF4 is mediated by histone deacetylation.

## Results

### ETT and ARF4 promote flower initiation with MP

**Fig. 1:**
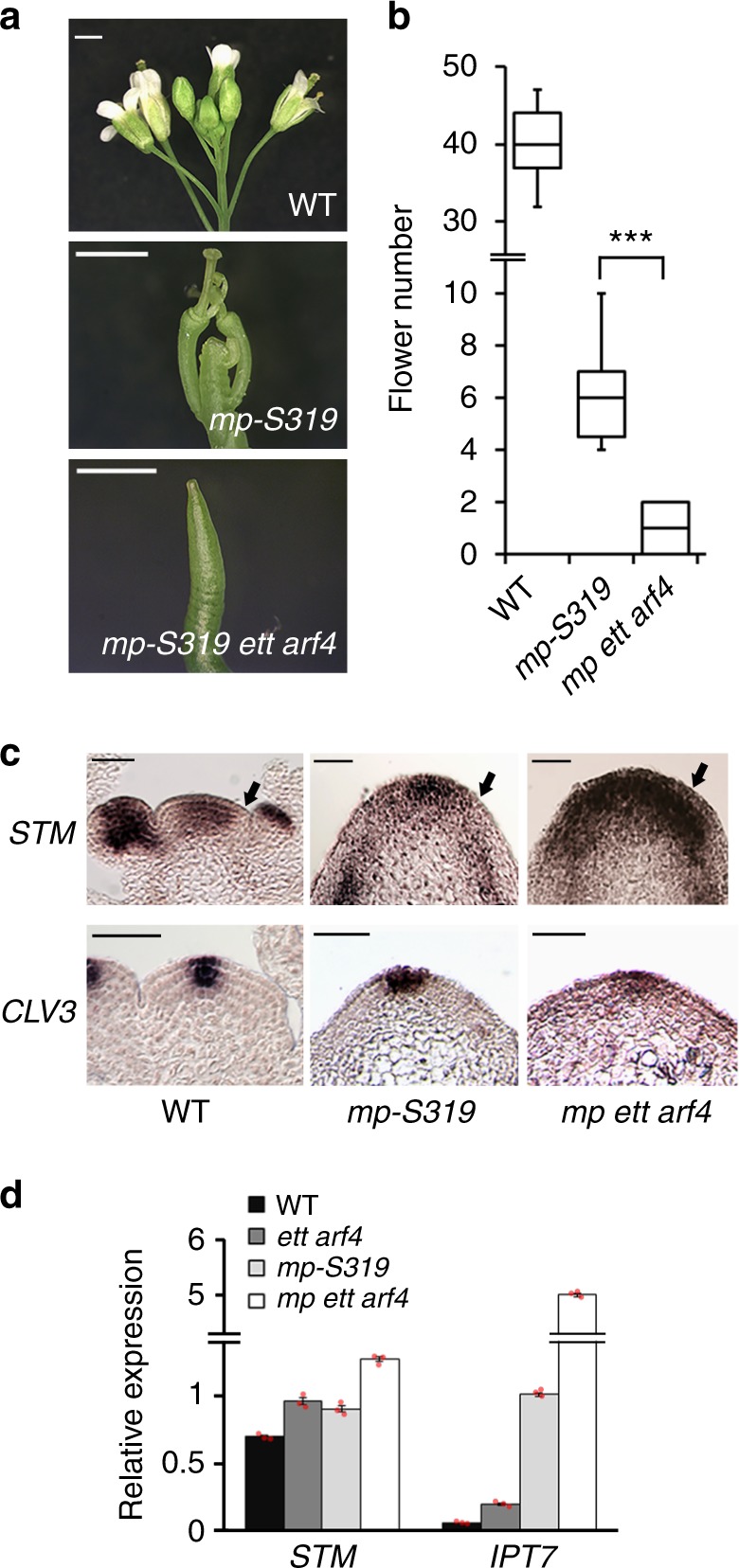
ETT and ARF4 contribute to initiation of reproductive primordia. **a**, **b** Flower initiation defect of the hypomorph *mp-S319* mutant compared to wild type (WT) and *mp-S319 ett arf4*. **a** Phenotype; Scale bar = 1 mm. **b** Quantification of flowers formed; ***P-value = 0.0001, one-tailed Mann–Whitney *U* test. *n* = 11 (WT) *n* = 11 (*mp-S319*) *n* = 9 (*mp-S319 ett arf4*). Box and whisker plot: Lower vertical bar = sample minimum. Lower box = lower quartile. Black Line = median. Upper box = upper quartile. Upper vertical bar = sample maximum. **c** Expression of the pluripotency genes *SHOOTMERISTEMLESS* (*STM*; top) and *CLAVATA3* (*CLV3*; bottom) in inflorescence apices assayed by in situ hybridization. The black arrow points to the region where *STM* expression is downregulated in the incipient primordium. Scale bar = 50 µm. **d** Relative expression of the *STM* class I KNOX gene and the STM target *IPT7* in trimmed inflorescence apices normalized over that of the *UBQ10* housekeeping gene. Shown are mean ± SEM of three experiments. Source data are provided as a Source Data file

To probe whether ETT and ARF4 contribute to flower initiation, we performed a genetic enhancer test using the hypomorphic *mp* allele *mp-S319*^27^. *mp-S319* has a T-DNA insertion in the second to last exon of *MP*^28^ and displays weaker phenotypes than *mp* null mutants, including partially compromised flower initiation (Fig. 1a, b)^10^. Loss of ETT and ARF4 activity in this background caused formation of naked inflorescence ‘pins’ that lack flowers (Fig. 1a, b). We also generated triple mutants between the MP interacting chromatin remodeler SYD and ETT/ARF4. Like *mp-S319 ett arf4, syd-5 ett arf4* mutants formed naked inflorescence pins (Supplementary Fig. 1a, b). These data suggest that the class B ARFs ETT and ARF4 promote flower initiation.

Stipules formed on *syd-5 ett arf4* inflorescence pins, pointing to increased meristematic activity^29,30^. Indeed, molecular characterization of the flower initiation defects of *syd-5 ett arf4* and *mp-S319 ett arf4* revealed a striking expansion of the *STM* expression into incipient reproductive primordia (Supplementary Fig. 1c, Fig. 1c). The expression domain of the shoot apical meristem stem cell marker *CLAVATA3* (*CLV3*) was not dramatically increased, suggesting that the expansion of the *STM* expression domain is not accompanied by an increase in the size of the *CLV3*-expressing stem cell pool. (Fig. 1c, Supplementary Fig. 1c). As in the wild type (WT)^25,26^, *STM* was downregulated at the flanks of the shoot apex of *mp-S319* and *ett arf4* mutants (Fig. 1c, Supplementary Fig. 2). Slightly elevated expression of *STM* was also detected by quantitative reverse transcriptase PCR (qRT-PCR) in entire *mp-S319 ett arf4* or *syd-5 ett arf4* inflorescences (Fig. 1d, Supplementary Fig. 1d). The observed increase in *STM* levels in *mp-S319 ett arf4* is probably an underestimate, because *mp-S319 ett arf4* mutants lack *STM* expressing flower meristems, in contrast to the other genotypes tested. We propose that the overexpression of *STM* in *mp-S319 ett arf4* is likely to be functionally important, because it triggered a marked increase in the expression of the STM target *IPT7*, a cytokinin hormone biosynthesis gene^23^ (Fig. 1d).

**Fig. 2:**
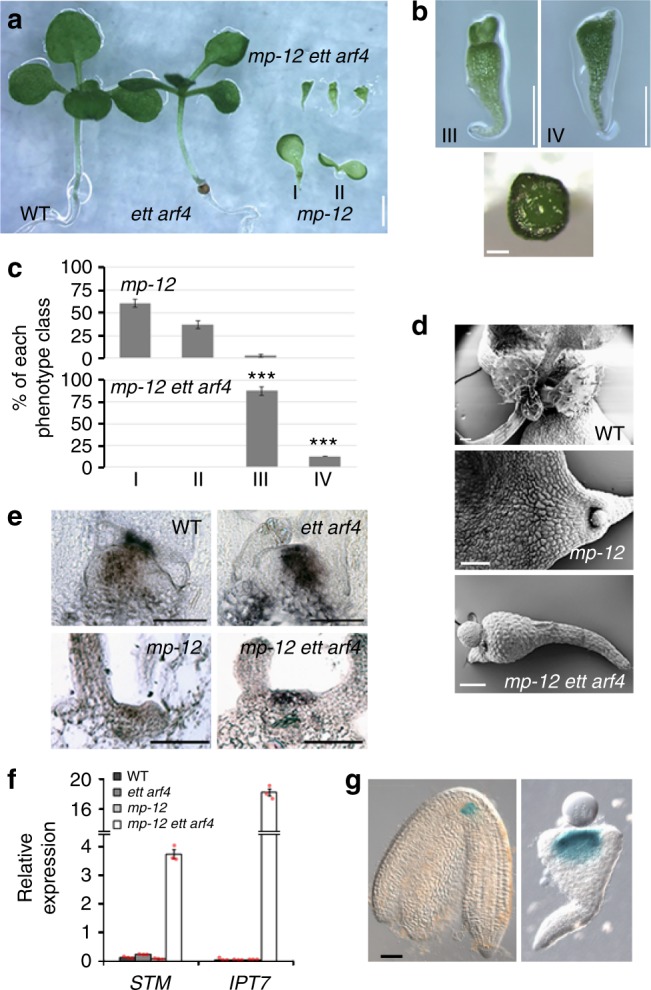
ETT and ARF4 act in a pathway parallel to MP. **a** Phenotypes of 10-day-old wild type (WT), *ett arf4*, *mp-12* null mutant and *mp-12 ett arf4* seedlings. Scale bar = 1 mm. Two phenotypic classes of *mp-12* seedlings are indicated (I, II). **b** Top: Close-up view of *mp-12 ett arf4* seedlings shown in **a**. Scale bar = 500 um. Two phenotypic classes of *mp-12 ett arf4* seedlings are indicated (III, IV). Bottom: Meristem of a class IV *mp-12 ett arf4* seedling viewed from above (Scale bar = 200 μm). **c** Number of *mp-12* or *mp-12 ett arf4* seedlings belonging to the phenotypic classes (I–IV) shown in **a** and **b**. The error bars are proportional to the standard error of the pooled percentage computed using binomial distribution^9^. ***P-value = 0.00001 for both *mp-12 ett arf4* III and IV class phenotype relative to *mp-12* class III and IV mutants; two-tailed Mann–Whitney *U* test. *n* = 114 and 51 for *mp-12* and *mp-12 ett arf4*. Source data are provided as a Source Data file. **d** Scanning electron microscopy images of representative 10-day-old WT, *mp-12* and class III *mp-12 ett arf4* seedlings. Scale bar = 200 μm. **e** *STM* expression patterns by in situ hybridization in 7-day-old seedlings. Scale bar = 50 μm. **f** Relative expression of *STM* and the STM target *IPT7* in 10-day-old seedlings normalized over that of *UBQ10*. Shown are mean ± SEM of three experiments. Source data are provided as a Source Data file. **g** Expression of *pSTM:GUS* in matureWT and *mp-12 ett arf4* embryos. Scale bar = 50 µm

To probe whether ETT and ARF4 act in parallel with MP, we generated triple mutants between the *mp-12* null mutant^31^ and *ett arf4*. While most of the plant body is elaborated post embryogenesis, the root, hypocotyl and embryonic leaves (cotyledons) are formed during embryo development^32,33^. *mp-12 ett arf4* mutants, like *mp*-*12* mutants, did not form roots (Fig. 2a, b). In addition, *mp-12 ett arf4* seedlings exhibited severe defects in cotyledon initiation and had enlarged shoot apical meristems (Fig. 2a–d). In situ hybridization in *mp-12 ett arf4* seedlings demonstrated an expansion of the *STM* expression domain into the region from where primordia initiate (Fig. 2e). qRT-PCR confirmed strongly increased expression levels of *STM* and *IPT7* in the triple mutant relative to the parental lines (Fig. 2f). An expanded *STM* expression domain was also apparent in mature embryos segregating in *mp-12/+ ett/+ arf4/-* plants that expressed a pSTM:GUS reporter^34^ (Fig. 2g). Based on these results, we conclude that ETT and ARF4 act in parallel with MP to promote organogenesis by repressing *STM* expression.

### *STM* downregulation is important for flower initiation

**Fig. 3:**
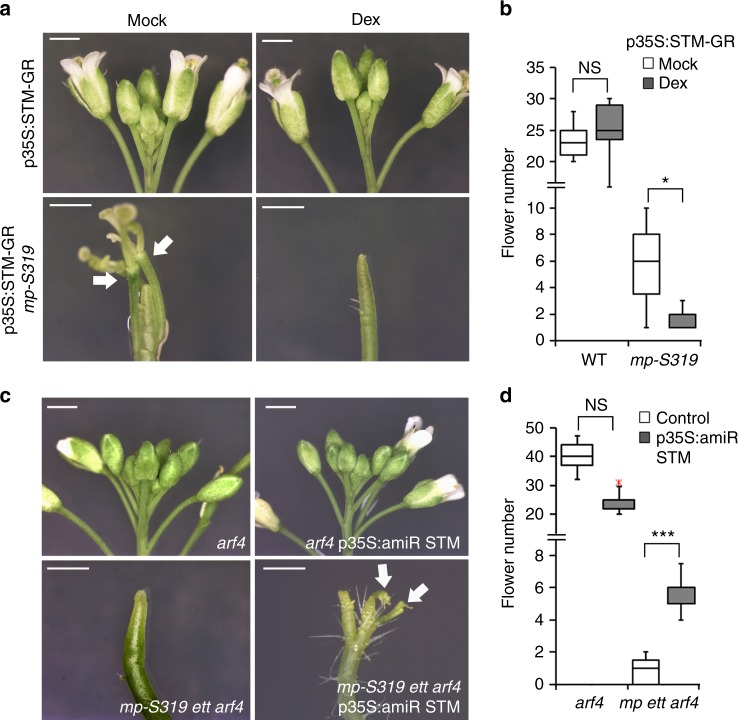
*STM* downregulation is important for flower initiation. **a**, **b** Primordium initiation defect of *mp-S319* compared to *mp-S319* without (Mock) and with conditional increase in STM activity upon activation of p35S:STM-GR with the synthetic steroid dexamethasone (Dex). **a** Phenotype; white arrow points to developing flowers. Scale bar = 1 mm. **b** Quantification of flowers initiated. Box and whisker plot: Lower vertical bar = sample minimum. Lower box = lower quartile. Black Line = median. Upper box = upper quartile. Upper vertical bar = sample maximum. NS *P*-value = 0.11, *P-value = 0.024 one-tailed Mann–Whitney *U* test. *n* = 15 and 11 (WT mock and dex) *n* = 8 and 5 (*mp-S319* mock and dex). Source data are provided as a Source Data file. **c**, **d** Primordium initiation defect of *mp-S319 ett arf4* compared to *mp-S319 ett arf4* plants in which *STM* expression is knocked down using an artificial microRNA, amiRSTM^35^. **c** Phenotype; white arrow points to developing flowers. Scale bar = 1 mm. **d** Quantification of flowers initiated. Box and whisker plot: Lower vertical bar = sample minimum. Lower box = lower quartile. Black Line = median. Upper box = upper quartile. Upper vertical bar = sample maximum. NS *P*-value = 0.99; ***P-value = 0.00001 one-tailed Mann–Whitney *U* test. *n* = 11(*arf4*), *n* = 13 (*arf4 p35S:amiRSTM*), *n* = 8 (*mp-S319 ett arf4*), *n* = 15 (*mp-S319 ett arf4 p35S:amiRSTM*). *arf4* is indistinguishable from the wild type^20^. Source data are provided as a Source Data file

To directly test for a biological role of *STM* in flower initiation, we used a steroid inducible version of STM, p35S:STM-GR^22^ (Supplementary Fig. 3a) to conditionally elevate *STM* levels in *mp-S319* mutant inflorescence meristems. Increased *STM* accumulation significantly enhanced the *mp-S319* flower initiation defect, causing formation of naked inflorescence pins (Fig. 3a, b). In agreement with the known positive feedback loop between STM and cytokinin^22,23^, treatment with cytokinin (zeatin) likewise enhanced the flower initiation defects of the hypomorph *mp-S319* mutant (Supplementary Fig. 4). On the other hand, introduction of a previously characterized artificial microRNA that specifically targets *STM*^35^ into the *mp-S319 ett arf4* triple mutant significantly rescued the floral initiation defect of the triple mutant (Fig. 3c, d) despite displaying only a partial decrease in *STM* accumulation (Supplementary Fig. 3b). The combined data suggest that downregulation of the *STM* pluripotency gene promotes reproductive primordium initiation.

### ETT directly and MP indirectly repress *STM*

**Fig. 4:**
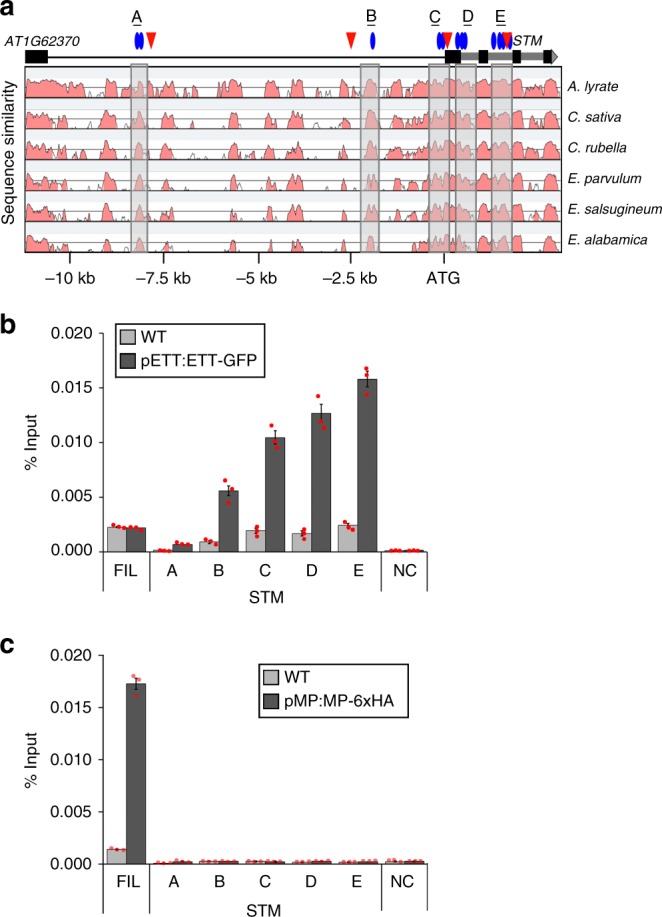
ETT binds the *STM* locus in vivo. **a** Phylogenetic shadowing of the *STM* locus. Top: five conserved domains (A–E) tested. Below: ARF binding sites and locus architecture. Blue ovals, conserved Auxin Response Factor core elements (TGTC); red triangles, ETT binding sites (TGTCAT);^36^ black boxes, exons; grey boxes, introns; black line, upstream intergenic region. Bottom: mVista plot. Grey shaded areas correspond to conserved regions tested. **b**, **c** Chromatin immunoprecipitation (ChIP) to test ETT (**b**) or MP (**c**) binding to conserved regions of the *STM* locus. TA3 served as negative control. An MP-bound region of the *FIL* locus^9^ served as positive control for MP ChIP (**c**) and as negative control for ETT ChIP (**b**). ChIP was performed under identical conditions in the wild type (WT). Shown are mean ± SEM of three experiments. Source data are provided as a Source Data file

The effect of ETT/ARF4 or MP on *STM* expression could be direct or indirect. To distinguish between these possibilities, we employed plant lines expressing biologically active genomic constructs for ETT-GFP^36^ or MP-6xHA^9^ (Supplementary Fig. 3c) to conduct chromatin immunoprecipitation (ChIP) followed by qPCR. We assayed binding to five evolutionarily conserved regions of the *STM* locus (Fig. 4a). ETT-GFP bound strongly and specifically to *STM*, with the strongest binding observed in the second intron, which has many putative ETT binding sites (Fig. 4a, b). MP did not associate with any of the regions of the *STM* locus tested (Fig. 4a, c), but did bind the previously identified direct target *FIL*^10^. Although our genetic analyses suggest that MP acts in parallel with ETT and ARF4 to repress *STM* expression, the chromatin immunoprecipitation data point to a possible indirect role of MP in this process. This conclusion was supported by examination of a second class I KNOX gene, *BREVIPEDICELLUS* (*BP*), whose expression is also elevated in *mp-S319 ett arf5* and *mp-12 ett arf4* mutants (Supplementary Fig. 5a). When we tested their association with this locus by ChIP-qPCR, we found that ETT, but not MP bound to the *BP* locus (Supplementary Fig. 5e–g).

### The MP target FIL represses *STM* together with ETT/ARF4

**Fig. 5:**
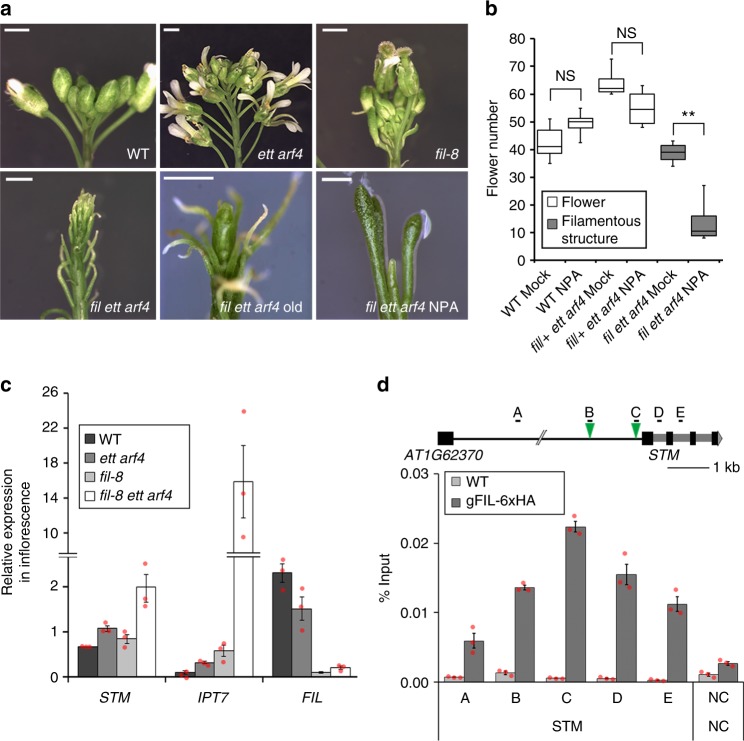
The MP target FIL promotes flower initiation together with ETT/ARF4. **a** Flower initiation phenotypes of wild type (WT), *ett arf4*, *fil-8*, young and old *fil-8 ett arf4* and auxin transport inhibitor (NPA) treated *fil ett arf4* plants. NPA (500 nM), was applied from 11 days of age until bolting. Scale bar = 1 mm. **b** Quantification of lateral organs formed in the absence and presence of NPA. Box and whisker plot: Lower vertical bar = sample minimum. Lower box = lower quartile. Black Line = median. Upper box = upper quartile. Upper vertical bar = sample maximum. *P*-values ( ± NPA): NS *P* = 0.11 (WT), NS *P* = 0.73 (*fil*/ + *ett arf4*) and *P = 0.006 (*fil ett arf4*), one-tailed Mann–Whitney *U* test. *n* = 7 (WT mock) *n* = 5 (WT NPA) *n* = 4 (*fil/* + *ett arf4* mock), *n* = 4 (*fil/* + *ett arf4* NPA) *n* = 5 (*fil ett arf4* Mock) *n* = 5 (*fil ett arf4* NPA). See supplementary Fig. 7a for images of mock and NPA treated control plants. Source data are provided as a Source Data file. **c** Relative expression of *STM*, *IPT7* and *FIL* normalized over that of *UBQ10* in the genotypes indicated. Shown are mean ± SEM of one representative of three experiments. Source data are provided as a Source Data file. **d** Chromatin immunoprecipitation (ChIP) to test FIL binding to conserved regions of the *STM* locus. Top: *STM* locus; green triangles, conserved FIL binding sites (AATNATAA)^41^. TA3 served as negative control. ChIP was performed under identical conditions in the WT. Shown are mean ± SEM of three experiments. Source data are provided as a Source Data file

We next asked whether one or several MP targets might act in concert with ETT and ARF4 to directly repress class I KNOX gene expression. One obvious candidate is the YABBY transcription factor FIL, a direct MP target with a role in flower initiation^9^. FIL has been shown to suppress class I KNOX gene expression in leaves^37^. In addition to its expression in the abaxial domain of developing leaves and flowers, *FIL* is expressed at the flanks of the shoot apex in the primordium founder cells from where primordia initiate^38–40^. We compared the accumulation of *FIL* mRNA with that of *ETT*, *ARF4* and *MP* in the reproductive shoot apex by situ hybridization and confirmed that all four genes are expressed in the primordium founder cells (Supplementary Fig. 6), the region where *STM* is depleted^26^. In agreement with a role for FIL in flower initiation, *fil ett arf4* triple mutant inflorescences displayed defects that ranged from initiation of filamentous structures in lieu of flowers to pins in older *fil ett arf4* inflorescences (Fig. 5a, b). Treatment with low doses of an auxin transport inhibitor caused formation of naked inflorescence pins in *fil ett arf4* plants, but not in any of the control genotypes (Fig. 5a, b, Supplementary Fig. 7). Furthermore, *fil ett arf4* mutant inflorescences also displayed increased expression of *STM*, *IPT7* and *BP* relative to the parental lines (Fig. 5c, Supplementary Fig. 5c). To test whether FIL directly regulates *STM* expression, we generated a biologically active, 6xHA-tagged genomic construct for FIL (Supplementary Fig. 3d). ChIP qPCR revealed that FIL bound to the *STM* locus, with the strongest binding observed at the proximal *STM* promoter (Fig. 5d), a conserved region with a FIL binding motif^41^. FIL also bound to the *BP* class I KNOX gene locus (Supplementary Fig. 5i). These data suggest that FIL directly represses class I KNOX genes including *STM* downstream of MP.

**Fig. 6:**
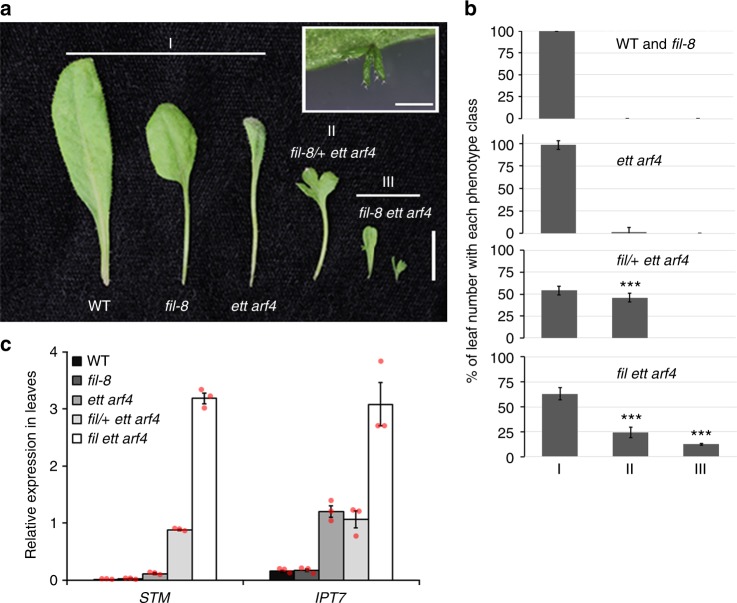
Leaf phenotypes of plants with reduced FIL, ETT and ARF4 activity. **a** Representative images of leaf phenotypes of wild type (WT), *fil-8*, *ett arf4*, *fil/+* *ett arf4* and *fil-8 ett arf4*. I-III indicate the different phenotypic classes observed. I simple leaves; II lobed leaves; III divided leaves and leaves bearing ectopic meristems (inset). Scale bars: 1 cm and 1 mm (inset). **b** Quantification of number of leaves in each phenotype class shown in **a**. The error bars are proportional to the standard error of the pooled percentage computed using binomial distribution *** *P* < 0.00001, two-tailed Mann–Whitney *U* test relative to *ett arf4*. *n* = 87 and 81 for WT and *fil-8*. *n* = 104, 148 and 64 for *ett arf4*, *fil*/+ *ett arf4* and *fil ett arf4*. Source data are provided as a Source Data file. **c** Relative expression of *STM* and *IPT7* in fully expanded adult leaves normalized over that of *UBQ10*. Shown are mean ± SEM of three experiments. Source data are provided as a Source Data file

A role for FIL and ETT/ARF4 in *STM* repression is further supported by the finding that *fil ett arf4* mutants also displayed leaf phenotypes characteristic of class I KNOX overexpressing plants^30^. These phenotypes were already apparent in *fil/+ ett arf4* mutants. Instead of the simple leaf typical of wild-type *Arabidopsis* plants, *fil/+* *ett arf4* leaves were lobed and divided (Fig. 6a, b). The leaf phenotype of the *fil ett arf4* triple mutants was even more severe. *fil ett arf4* mutants formed deeply divided leaves or leaves bearing leaflets with ectopic meristems (Fig. 6a, b). Moreover, leaves of *fil/+* *ett arf4* and of *fil ett arf4* mutants had increased *STM*, *IPT7* and *BP* expression (Fig. 6c; Supplementary Fig. 5c). The haploinsufficiency of the *fil/+* *ett arf4* mutant suggests that FIL and ETT or ARF4 may form a complex. Indeed, FIL was identified as an ETT interacting protein in a recent yeast-two-hybrid screen^36^. We confirmed that FIL interacts with ETT and ARF4 by yeast-two-hybrid tests, bifluorescence molecular complementation, and co-immunoprecipitation (Supplementary Fig. 8). The combined data implicate a YABBY/ARF complex in direct repression of *STM* during reproductive primordium initiation and formation of simple leaves in *Arabidopsis*.

### YABBY/ARF complex silences *STM* via histone deacetylation

**Fig. 7:**
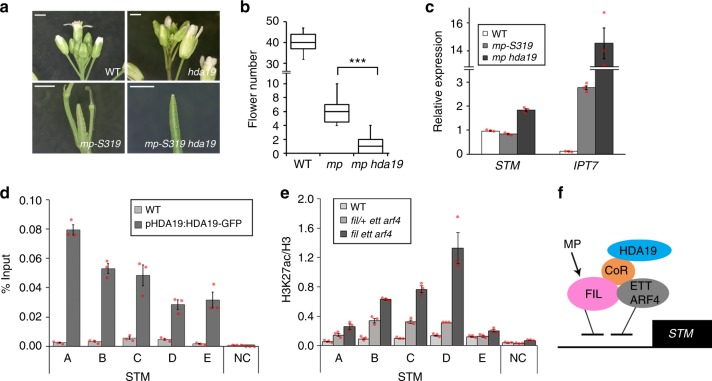
*STM* repression by FIL and ETT/ARF4 relies on reduced histone H3 acetylation. **a**, **b** Flower initiation phenotypes of wild type (WT), *hda19*, the hypomorph *mp-S319* mutant and *mp-S319 hda19*. **a** Phenotype; Scale bar = 1 mm. **b** Quantification of flowers formed; ***P = 0.00024, one-tailed Mann–Whitney *U* test. *n* = 11 (WT) *n* = 11 (*mp-S319*) *n* = 9 (*mp-S319 hda19*). **c** Relative expression of *STM* and *IPT7* in trimmed inflorescences normalized over that of *UBQ10*. Shown are mean ± SEM of three experiments. **d** Chromatin immunoprecipitation (ChIP) to test HDA19 binding to conserved regions of the *STM* locus in inflorescences. *TA3* served as negative control. ChIP was performed under identical conditions in the WT. Shown are mean ± SEM of three experiments. **e** Anti-histone 3 lysine 27 acetylation (H3K27ac) compared to H3 ChIP at the *STM* locus in expanded leaves of WT, *fil/+* *ett arf4* and *fil ett arf4*. *TA3* served as negative control. ChIP was performed under identical conditions in the WT. Shown are mean ± SEM of three experiments. **b**–**e** Source data are provided as a Source Data file. **f** Model for the repression of *STM* in incipient reproductive primordia. See text for details

Finally, we investigated how the YABBY/ARF complex represses *STM*. Histone deacetylation leads to repression of gene expression in the context of chromatin^42^. ETT, ARF4 and FIL interact with different transcriptional co-repressor complexes that all recruit the histone deacetylase HDA19^43–50^. Combined yeast-two-hybrid, in planta bifluorescence molecular complementation, and co-immunoprecipitation analyses confirmed an association of HDA19 with the ETT/ARF4 FIL complex (Supplementary Fig. 8). We therefore tested for a biological role of HDA19 in initiation of reproductive primordia. *mp-S319 hda19* double mutants had significantly enhanced flower initiation defects relative to the parental lines (Fig. 7a, b). The naked pin inflorescence phenotype of *mp-S319 hda19* was accompanied by increased expression of *STM*, *IPT7* and *BP* (Fig. 7c, Supplementary Fig. 5e). Treatment with histone deacetylase inhibitors also significantly enhanced the flower initiation defect of *mp-S319* (Supplementary Fig. 9). To further probe the biological role of HDA19 in initiation of reproductive primordia, we generated double mutants between HDA19 and the SWI/SNF chromatin remodeler SYD, which acts together with MP to upregulate genes that promote flower initiation^9^. Like *mp-S319 hda19*, *syd-5 hda19* inflorescences formed inflorescence pins and initiated significantly fewer flowers than the parental lines (Supplementary Fig. 1). In addition, *syd-5 hda19* inflorescences displayed elevated *STM* and *ITP7* expression (Supplementary Fig. 1).

To assess whether the histone deacetylase HDA19 binds to class I KNOX loci, we performed ChIP-qPCR using pHDA19:HDA19-GFP^9^ plants. HDA19 associated strongly with multiple regions of the *STM* and *BP* loci (Fig. 7d, Supplementary Fig. 5j). If FIL and ETT/ARF4 repression of *STM* is mediated by histone deacetylation, the triple mutant should display increased histone acetylation at the class I KNOX gene loci. We therefore tested for an increase in H3K27 acetylation in *fil ett arf4* mutants compared to parental lines. H3K27ac accumulation relative to that of H3 was elevated at both the *STM* and *BP* loci in *fil ett arf4* mutants (Fig. 7e, Supplementary Fig. 5). H3K27ac/H3 accumulation correlates with *STM* expression levels in different plant tissues (Supplementary Fig. 10). The combined data point to a role of FIL, ETT and ARF4 in *STM* and *BP* silencing by generating a repressive chromatin state via histone deacetylation. They further reveal that *STM* downregulation is important for primordium founder fate and that the ‘repressive’ class B ARFs ETT and ARF4 cooperate with the ‘activating’ class A ARF MP to silence *STM* together with HDA19 through a YABBY/ARF co-repressor complex (Fig. 7f).

## Discussion

Organogenesis is critical to establish the body plan of higher eukaryotes. Here we investigate the initiation of reproductive primordia that give rise to flowers, plant organs critical for reproductive success. Prior studies uncovered the gene expression programs activated by MP and the mechanism for this activation in response to auxin accumulation in the founder cells of reproductive primordia^9,10^. Downregulation of expression of the pluripotency gene *STM* has been observed in the primordium initials, transit amplifying cells that have perceived an auxin maximum^25,26^. Using complementary gain- and loss-of-function approaches, we show here that this *STM* downregulation is important for primordium initiation (Fig. 3). We further show that STM downregulation is mediated by MP indirectly, via its target FIL and – in a parallel pathway – by a pair of repressive ARFs, ETT and ARF4 (Figs. 1, 2, 4, 5). Finally, FIL and the class B ARFs jointly recruit the histone deacetylase HDA19 to silence *STM* (Fig. 7f). Removal of acetyl groups from lysine on histone H3 tails promotes chromatin compaction, a chromatin environment unfavorable for transcription^42^. A second class I KNOX gene, *BP*, is regulated in much the same way as *STM* (Fig. S5). On the basis of prior studies, *BP* downregulation also contributes to reproductive primordium initiation^51^.

*STM* is known to be a target of Polycomb Repressive Complex 2 (PRC2)^52–54^. In our genetic enhancer tests, we did not observe enhanced flower initiation defects when we combined *mp-S319* with a PRC2 mutant (Supplementary Fig. 11). Polycomb repression frequently drives long-term silencing^55^ and downregulation of *STM* in the reproductive primordium is not permanent. Once flowers have reached stage 2 of development, *STM* is upregulated when the floral meristem forms^26^.

It has been proposed that class B ARFs may inhibit activity of class A ARFs by competing for binding sites at the same target genes^56^. We show here that class B and class A ARFs can act cooperatively in the same pathway by directly repressing and activating different classes of genes (this study and references^9–13^). Evidence is accumulating for unique DNA sequence binding preferences of ETT and MP^41,57–59^. It is likely that multiple different types of interactions between the evolutionarily conserved classes of ARFs^14,60,61^ enable unique transcriptional and developmental outcomes in response to a single hormonal cue, auxin. Additional specificity is provided by ARF interactions with other classes of transcription factors, such as the YABBY transcription factor FIL we identify here.

Auxin promotes *FIL* accumulation in incipient reproductive primordia by triggering ubiquitin-mediated degradation of Aux/IAA proteins complexed with MP, thus enabling MP to recruit chromatin remodelers to activate *FIL* expression^8,9^. How auxin modulates ETT/ARF4 accumulation or activity to promote flower initiation is not clear. In a pathway parallel to MP, auxin may regulate *ETT* and *ARF4* message abundance transcriptionally or post transcriptionally via the transacting siRNAs that target these class B ARFs^18^. Alternatively, ARF4 activity may be inhibited by Aux/IAA proteins in conditions of low auxin. In this scenario, activity of ETT, which lacks the Aux/IAA protein interacting domain, could be blocked by Aux/IAA proteins as a DNA-binding domain mediated heterodimer with other ARFs, such as ARF4^16,58^. Since ETT activity is modulated by changes in auxin concentration^36,59^, auxin might instead control ETT interaction with FIL and/or HDAC-containing co-repressor complexes.

In summary, our study implicates epigenetic silencing of a pluripotency factor in initiation of reproductive primordia, and elucidates the mechanism for this silencing. Our findings moreover suggest that in plants, like in animals^62^, organogenesis requires both activation of lineage-specific gene expression programs and silencing of pluripotency genes.

## Methods

### Plant materials and treatments

Plants were grown at 22 °C in long-day conditions (16 h day/8 h dark) in soil or on ½ Murashige and Skoog (MS) plates^63^. All mutations were in the Columbia accession unless otherwise indicated. Mutant alleles described before include *ett-7*^43^*, arf4-2* (SALK_070506)^20^*, mp-S319*^27^, *hda19-1*^64^*, mp-12 (SALK_049553*)^31^*, clf*-*28*^65^, pETT:ETT-GFP *ett -3*^36^, *syd-5*^66^ and pHDA19:HDA19-GFP^9^. *fil-8*^67^ is in the L*er* background and was outcrossed to Columbia more than three times. *fil_049 (WiscDsLox367E6_049*) was obtained from the Arabidopsis Biological Resource Center.

For activation of p35S:STM-GR in *mp-S319*, plants were treated with 1 µM dexamethasone (DEX, Sigma) plus 0.015% Silwet from 12 days of age onwards, the stage when the first reproductive primordium initiates^68^. Treatment was continued for 9 days, because new reproductive primordia initiate continuously and the synthetic steroid rapidly decays in plants^69^. For expression studies, 9-day-old MS agar-grown seedlings were sprayed with 10 µM Dex and seedlings were harvested after 4 h. Mock treatment consisted of 0.1% DMSO and 0.015% Silwet. For the cytokinin treatment, *mp-S319* inflorescences were treated with 1 µM cytokinin (trans-Zeatin, Sigma) in 0.015% Silwet from 11 days of age onwards every 2 days, for a total of eight treatments. Mock treatment consisted of 0.1% DMSO and 0.015% Silwet. For deacetylase inhibitor treatment, soil-grown plants were sprayed with 5 µM of Trichostatin A (TSA, Sigma) plus 0.015% Silwet or with 50 µM of Suberoylanilide hydroxamic acid (SAHA, Sigma) plus 0.015% Silwet every 3 days from 11 days of age onwards for a total of six treatments. After manifestation of pin inflorescence phenotypes, spraying was resumed once weekly for 2 weeks. Mock treatment consisted of 0.1% DMSO plus 0.015% Silwet. For NPA treatment, *fil ett arf4* inflorescences were treated with 500 nM of NPA plus 0.015% Silwet every 2 days on from 11 days of age onwards for a total of total eight treatments. Mock treatment consisted of 0.05% DMSO plus 0.015% Silwet. Because treated plants are less vigorous and make fewer flowers then untreated plants (compare WT in Fig. 1b to Fig. 3b), phenotypes of treated plants were always compared to treated control plants. The number of flowers formed was counted in 55–65-days-old plants, once development had ceased, to ensure an accurate count of all flowers or filamentous structures initiated.

### Transgenic plants

p35S:STM-GR^70^ was transformed into *mp-S319*/+. In the T1 generation, plants heterozygous for *mp-S319* and hemizygous for STM-GR were selected by genotyping and growth on selective medium, respectively. In the T2 generation, seedlings were germinated on selective medium to recover plants hemi or homozygous for the transgene and transferred to soil. Plants homozygous for WT or *mp* were identified by genotyping. Steroid treatment and phenotypic analyses were conducted in the T2 generation on WT and *mp-S319* siblings.

p35S:amiR STM^35^ was transformed into *mp-S319/+* *ett-7/+ arf4-2*. In the T1 generation, plants carrying the transgene (amirSTM) were identified by BASTA treatment and plants heterozygous for *mp* and *ett* by genotyping. In the T2 generation, T1 progeny was sprayed with BASTA to identify plants hemi or homozygous for amiRSTM. *mp ett arf4* triple mutants were identified visually (they form very few leaves) and confirmed by genotyping. amiRSTM *arf4* plants were identified by visual inspection and genotyping from the same T1 progeny. Phenotypic analyses were conducted in the T2 generation on *arf4* and *mp-S319 ett arf4* siblings. The *arf4* single mutant is indistinguishable from the WT^20^.

To generate ETTr, the tasi-RNA target sites of ETT^19^ were mutagenised by site-directed mutagenesis using primer sets previously described^19^. The ETTr fragment was amplified by PCR and cloned into pENTR/D Topo (Thermo Fisher Scientific). To generate ARF4r, the full length ARF4 coding sequence was amplified and cloned into pENTR/D TOPO. 266 base pairs including the sequence with the mutated tasi-RNA target sites^19^ in ARF4 was synthesized as a gBlock Gene Fragment (Integrated DNA Technologies). The endogenous sequence was replaced by the synthesized gBlock in pENTR/D TOPO using Gibson assembly with the Gibson assembly master mix (New England BioLabs). For BiFC assays, ETTr, ARFr, as well as FIL and HDA19 in pENTR/D TOPO (see Supplemental Table 1 for primer information) were recombined to pUC-SPV-NE^GW^ and pUC-SPV-CE^GW^ ^71^ for BiFC. pMP:MP-6xHA^9^ was transformed into *mp-12*/ + . Lines which showed full phenotypic rescue were selected for ChIP. To construct gFIL-6xHA, the genomic locus of FIL, including the 5780 bp upstream intergenic region plus the coding region with introns was amplified as a single module and assembled together with 6xHA and tNOS terminator using the BsaI-HF enzyme (New England BioLabs) in an entry Gateway compatible vector. Following LR reaction (Invitrogen), the pFIL:FIL-6xHA-tNOS fragment was recombined into a modified Gateway-compatible version of the pPZP222 vector. To increase the cut-ligate reaction efficiency, site-specific mutagenesis was used to mutate two endogenous BsaI site in the FIL locus. The final destination vectors were transformed into in the *fil_049* mutant background. Constructs used for stable plant transformation were transformed into *Agrobacterium tumefaciens* strain GV3101 by electroporation, followed by plant transformation by floral dip.

### Phylogenetic shadowing

Genomic sequence covering from AT1G62370 to the *STM* 3′UTR was used as a query for finding sequences of closely related species. The genomic sequences of *Brassicaceae* species were obtained by running BLASTn discontinuous megablast. The mVISTA program (http://genome.lbl.gov/vista/index.shtml) was employed to identify regions of conservation. Clustal omega (https://www.ebi.ac.uk/Tools/msa/clustalo/) was used to identify conserved cis motifs in the conserved regions of the *STM* locus.

### Chromatin immunoprecipitation

ChIP was performed as described in our detailed published protocol^72^. For inflorescence ChIP, ~0.5 g of young inflorescences from plants with 0~5 cm bolts were harvested. All flowers from stage 6 onwards were removed. Anti-GFP (Thermo Fisher Scientific, A11122, Lot 2015992; 1:200 dilution) and Anti-HA (Roche, 12CA5, Lot 15782900; 1:40 dilution) were used for IP. Antibody quality was validated by confirming published ChIP data for LFY-GFP^73^ and FIE-HA^71^. In addition, Anti-H3K27ac antibodies (Abcam, ab4729, lot GR3216173-1; 1:500 dilution), anti-H3K9ac antibodies (Active Motif, 39137, lot 9811002; 1:500 dilution) and anti-H3 antibodies (Abcam, ab1791, lot GR3198255-1, 1:500 dilution) were used for IP. The antibodies were validated by the manufacturers. For ChIP in wild-type leaves and *ap1 cal* inflorescences, ~2 g of fully expanded Columbia leaves and 0.5 g of *ap1-1 cal-1*^74^ inflorescence tissue was collected and fixed for analysis. Antibody amounts were 5 µg for anti GFP and HA, and 2 µg for anti H3, H3K27ac and H3K9a. The *TA3* retrotransposon (At1g37110) served as negative control locus (NC)^9^. The *LFY* locus region e (*LFYe*)^10^ served as positive control for MP ChIP and as a negative control for ETT ChIP. Throughout, ChIP was also performed using the same antibodies in the WT to control for nonspecific DNA enrichment. To estimate the DNA enrichment, the qPCR value of the ChIP product was normalized over input DNA. Primer sequences are listed in Supplementary Table 1.

### Expression analysis

In situ hybridization of *STM*, *CLV3*, *MP* and *ETT* was performed as described in our published protocol^75^ with minor modifications. The antisense probes for *STM*, *FIL* and *ARF4* were generated using in vitro transcription from the full- length *STM* (1149 base pair)*, FIL* (690 base pairs) and *ARF4* (2367 base pairs) coding sequences cloned into pGEM-T (Promega). The full-length (516 base pair) *CLV3* coding sequence and 1273 base pair of the *ETT* coding sequence (551–1824 base pairs) were cloned into pGEM-T easy for probe synthesis. The *MP* probe has been described^10^. Inflorescence tissue was harvested at 21–28 days of age for WT and *ett arf4* and at 28 days of age for genotypes showing pin phenotypes (*mp-S319* and *mp-S319 ett arf4*). Seedling tissue was grown on MS agar medium and harvested at 10 days of age. Pictures were taken of 8 μ sections using a brightfield microscope (Olympus BX51).

For qRT-PCR expression analysis from 1–5 cm bolt inflorescences and fully expanded adult leaves, RNA was extracted by TRIzol (Thermo Fisher Scientific) and further purified using the RNeasy mini kit (Qiagen). First strand cDNA was synthesized using the superscript III kit (Thermo Fisher Scientific) from 1 µg of RNA. Quantitative real time PCR was performed using Power SYBR Green master mix (Thermo Fisher Scientific). Relative expression value was determined using a cDNA standard curve and normalized over that of the housekeeping gene *UBQ10* (AT4G05320).

### Protein interaction

For test of interaction between ETT, ARF4, FIL and HDA19 in yeast, full length clones for all four proteins, as well as ETT-N (amino acids 1–389) and ETT-C (amino acids 390–608) were cloned into pENTR/D TOPO and recombined into pDEST22 and pDEST32. These constructs were co-transformed into competent AH109 yeast cells using the Frozen-EZ Yeast Transformation II Kit (Zymo research). To examine yeast growth, serially-diluted yeast cells were spotted on media lacking Leucine and Tryptophan. To test for interactions, yeast cells were spotted on media lacking Leucine, Tryptophan and Histidine and containing 3-amino-1,2,4-triazole (3-AT) as competitive inhibitor. 3-AT concentrations used were 1 mM and 0.1 mM. Pictures were taken after 3 days of growth.

Bimolecular fluorescence complementation (BiFC) in *Arabidopsis* leaf protoplasts was performed as previously described^76^. Only protoplasts prepared and transfected at the same time were used for direct comparison. Three-week-old soil-grown Columbia plants were either sprayed with 10 µM of indole-3-acetic acid (IAA, Sigma) plus 0.015% silwet or with 0.01% DMSO plus 0.015% Silwet nine hours prior to protoplasting. To avoid message depletion by tasiRNAs, tasiRNA resistant versions of ETT (ETTr) and ARF4r version were employed. Protoplasts (70–280) were counted per replicate and sample. Confocal imaging was conducted using a Leica TCS SP8 confocal microscope and a 40X water immersion objective.

For co-immunoprecipitation, ETT-Myc, ARF4-FLAG, ARF4-Myc, HDA19-FLAG and HDA19-Myc were generated using the same entry vectors as for BiFC (above) following recombination into pGWB12 and pGWB18^77^ or FLAG-tag and 4xMyc-tag fusion, respectively. ETT-FLAG was generated using golden gate cloning and a previously described L0 clone for ETT^36^, combined with the p35S promoter (AddGene #50266), the C-terminal 3xFLAG epitope (AddGene #50308), and the Nos-terminator (AddGene #50339) into the L1 (AddGene #48000)^78^. The epitope-tagged proteins were transiently expressed in 4-week-old *N. benthamiana* leaves by means of infiltration with an *Agrobacterium* suspension (OD600 = 0.5). Two days after infiltration, 1 g of leaf tissue was collected and ground in liquid nitrogen. All subsequent steps were carried out at 4 °C. The ground powder was homogenized for 30 min in two volumes of extraction buffer (10% glycerol, 25 mM Tris-HCl pH 7.5, 1 mM EDTA, 150 mM NaCl, 0.15% NP-40, 1 mM PMSF, 10 mM DTT, 2% Polyvinylporrolidone, 1x cOmplete Mini tablets EDTA-free Protease Inhibitor Cocktail (Roche)). The homogenized samples were cleared by centrifugation at 14,000 × *g* for 10 min. The supernatant was transferred to 2 ml microcentrifuge tubes and cleared for another 5 min by centrifugation 14,000 × *g*. Next, the cleared lysate was transferred to fresh 2 ml microcentrifuge tubes and incubated for 2 h with 20 µl anti-FLAG M2 magnetic beads (SIGMA-ALDRICH, M8823; lot: SLB2419). The beads were subsequently washed five times with IP buffer (10% glycerol, 25 mM Tris-HCl pH 7.5, 1 mM EDTA, 150 mM NaCl, 0.15% NP-40, 1 mM PMSF, 1 mM DTT, 1x cOmplete Mini tablets EDTA-free Protease Inhibitor Cocktail (Roche)). Proteins were eluted by adding 80 µl 1x SDS loading buffer followed by an incubation at 95 °C for 10 min. The eluate was analysed by western blot using an anti-FLAG antibody (M2, Abcam, ab49763, lot: GR3207401-3) or an anti-Myc antibody (9E10, Abcam, ab62928, lot: GR3208762-2). Both antibodies were used at a 1:10000 dilution. The antibodies were validated by the manufacturer.

### Scanning electron microscopy

For scanning electron microscopy, 10-day-old seedlings were fixed overnight in FAA (formaldehyde 3.7%, glacial acetic acid 5%, ethanol 50%) and dehydrated in a serial dilution of ethanol. The samples were critical point dried, mounted and gold coated. Images were taken with a Zeiss Supra 55VP Field emission scanning electron microscope using an acceleration voltage of 3 kV.

### STM reporter studies

Late bent cotyledon stage embryos from *mp-12/+ett/+ arf4/-* plants homozygous for a pSTM:GUS reporter^30^ were dissected from mature siliques, and stained in restrictive GUS staining solution (50 mM Sodium Phosphate Buffer pH 7.2, 0.2% Triton X-100, 2 mM Potassium Ferrocyanide, 2 mM Potassium Ferricyanide, and 2 mM X-Gluc in N, N-dimethylformamide) for 5 h at 37 °C. Embryos were mounted in concentrated Hoyer’s solution (3.75 g of Arabic gum, 2.5 ml of glycerol, 50 g of Chloral hydrate adjusted with distilled water to 50 ml final volume), cleared overnight, and observed with a Leica DMR6000 microscope with Nomarski optics at ×10 magnification. Homozygous *mp ett arf4* embryos were identified by comparing segregation ratios of phenotypes between *mp-12/+* and *mp-12/+* *ett/+* *arf4/-* plants.

### Statistical analyses and replication

Throughout, error bars shown represent the standard error of the mean for all numerical values. For frequencies, the error bars are proportional to the standard error of the pooled percentage computed using binomial distribution^9^. For qRT-PCR and ChIP-qPCR, one representative of three experiments is shown. Box and whisker plots: Lower vertical bar = sample minimum. Lower box = lower quartile. Black Line = median. Upper box = upper quartile. Upper vertical bar = sample maximum. A nonparametric Mann Whitney *U* test was used for phenotypic data, which frequently are not normally distributed. Unpaired *t*-tests were used for qRT-PCR and ChIP-qPCR data, which were normally distributed based on Kolmogorov–Smirnov tests. We employed one-tailed tests to examine differences between groups in a specific direction, and two-tailed tests to probe differences between groups in any direction. Sample size was determined by expedience and based on prior studies that found significance using similar samples sizes^9,10,71,73^.

### Reporting summary

Further information on experimental design is available in the Nature Research Reporting Summary linked to this article.

## Supplementary information

Supplementary Information

Reporting Summary

Source Data

## Data Availability

The authors declare that the data supporting the findings of this study are available within the paper and its supplementary information files. The data underlying Fig. 2c, Fig. 6b and Supplemental Fig. 8c as well as *P*-values for all figures provided as a source data file.
