# Supplementary material for: Auxin Response Factors promote organogenesis by chromatin-mediated repression of the pluripotency gene SHOOTMERISTEMLESS
Source: Nat Commun. 2019 Feb 21;10:886. doi: 10.1038/s41467-019-08861-3 (PMC6385194; doi:10.1038/s41467-019-08861-3)
Supplement: Supplementary file 1 — Supplementary Information [file 41467_2019_8861_MOESM1_ESM.pdf]

SUPPLEMENTARY INFORMATION

Auxin Response Factors promote organogenesis by chromatin-mediated repression of the pluripotency gene *SHOOTMERISTEMLESS*

Yuhee Chung *et al.*

Supplementary Figures

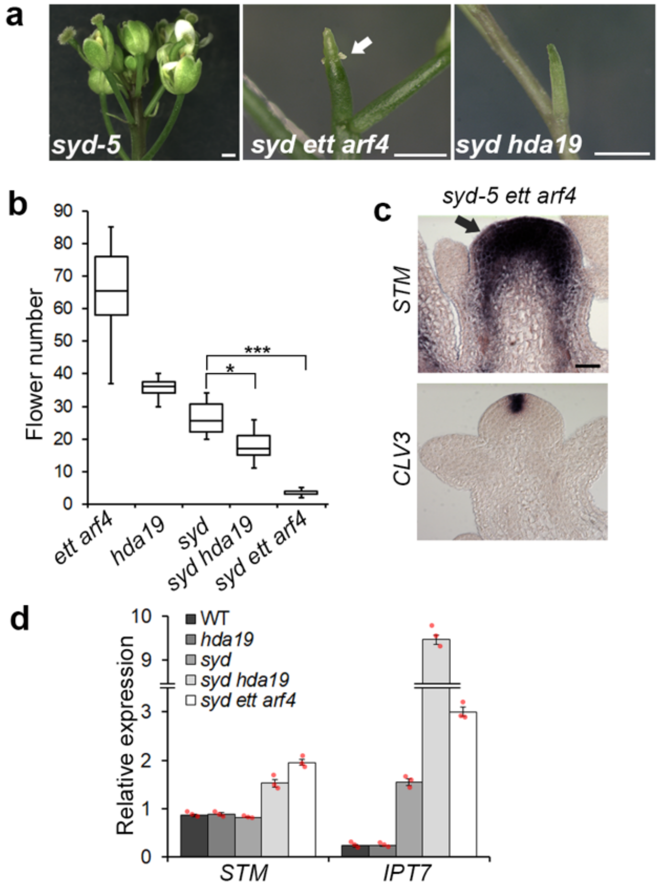

Supplementary Fig. 1 Flower initiation defects in *syd ett arf4* and *syd hda19* mutants

a Phenotype of the *syd-5* null mutant, *syd*-5 *ett arf4* and *syd-5 hda19* inflorescences. Scale bar = 1mm. the white arrow points to a stipule.

b Quantification of flowers initiated in the genotypes shown in **a.** Box and whisker plot: Lower vertical bar= sample minimum. Lower box=lower quartile. Black Line= median. Upper box= upper quartile. Upper vertical bar= sample maximum. *** P-value = 0.00001, * P-value = 0.014, n= 10, 19, and 5 for *syd*, *syd ett arf4 and syd hda19*.

c Expression of the pluripotency genes *SHOOTMERISTEMLESS* (*STM*; top) and *CLAVATA3* (*CLV3*; bottom) in inflorescence apices assayed by *in situ* hybridization. The black arrow points to the region where *STM* expression is downregulated in the incipient primordium. Scale bar = 30 μm.

d Relative expression of the *STM* class I KNOX gene and the STM target *IPT7* in trimmed inflorescence apices normalized over that of the *UBQ10* housekeeping gene. Shown are mean +/- SEM of three experiments.

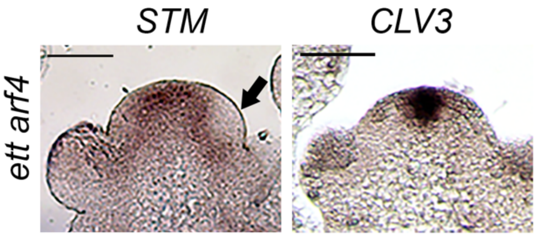

Supplementary Fig. 2 Expression of pluripotency genes in *ett arf4*
Expression of *SHOOTMERISTEMLESS* (*STM*; left) and *CLAVATA3* (*CLV3*; right) in *ett arf4* inflorescence apices assayed by *in situ* hybridization. The black arrow points to the region where *STM* expression is downregulated in the incipient reproductive primordium. Scale bar = 50 µm. For a picture of the *ett arf4* inflorescence phenotype see Fig. 5 a.

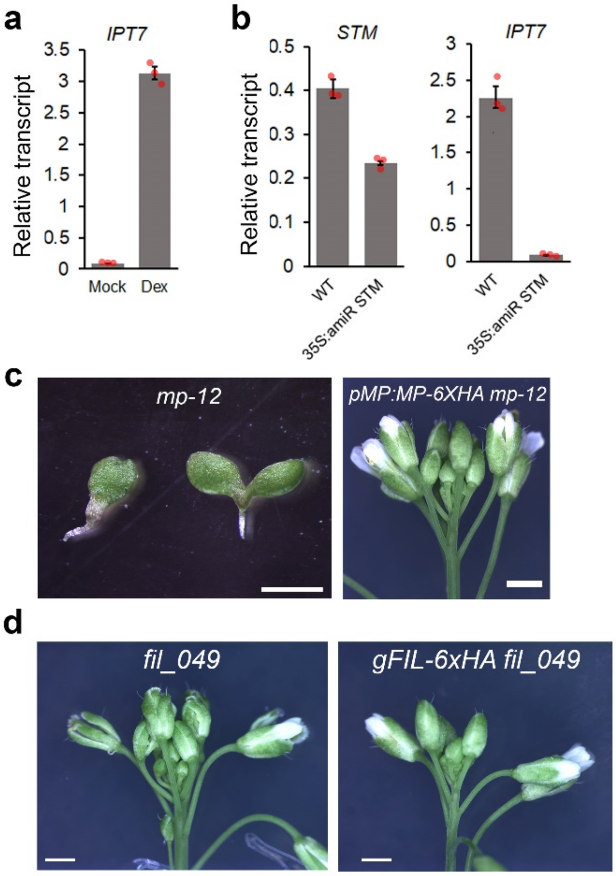

Supplementary Fig. 3 Test of transgenic plant lines generated

a, b Test of activity of p35S:STM-GR and amiRSTM. (**a**) Upregulation of the STM target *IPT7*, as previously reported[1], four hours after steroid activation (1 µM dexamethasone) of STM-GR in inflorescences. (**b**) Moderate reduction of *STM* levels and strong reduction of the STM target *IPT7* in *mp ett arf4* mutant inflorescences expressing a previously described artificial microRNA, *amiRSTM*[2]. All expression values were normalized of that of *UBQ10*. Shown are mean+/-SEM of three experiments.

c, d Rescue lines used for ChIP. The epitope tagged constructs for MP (pMP:MP-6XHA) (**c**) and FIL (gFIL-6XHA) (**d**) fully rescue the morphological defects of the respective mutants. Scale bar: 1 mm.

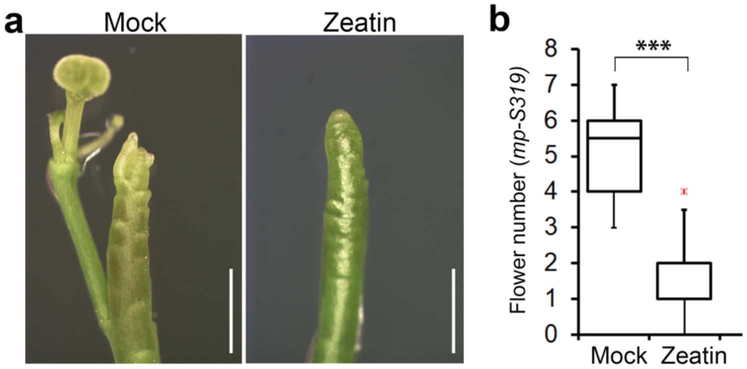

Supplementary Fig. 4 Cytokinin treatment enhances the organogenesis defect of the hypomorph *mp-S319* mutant

a, b Treatment of *mp-S319* with 1 µM Cytokinin (trans-Zeatin) strongly enhanced the flower initiation defect. Scale bar = 1 mm (**a**) Representative phenotype images. (**b**) Quantification of flowers initiated genotypes shown in **a.** Box and whisker plot: Lower vertical bar= sample minimum. Lower box=lower quartile. Black Line= median. Upper box= upper quartile. Upper vertical bar= sample maximum. *** P=0.00017, one-tailed Mann-Whitney U test. n=12 (mock), n=9 (Zeatin).

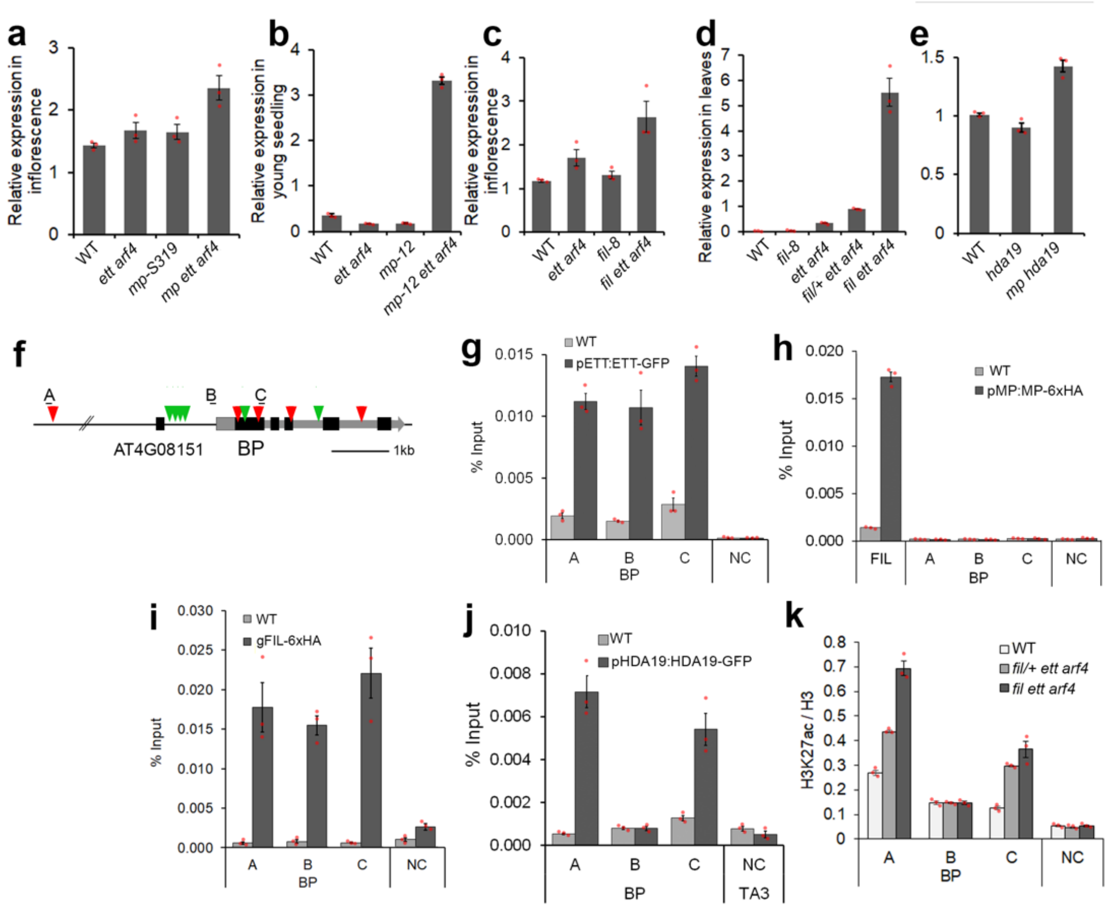

Supplementary Fig. 5 Expression of class I KNOX gene *BP*, and chromatin binding at *BP*
a-e. Relative to parental lines, *BP* expression is elevated in *mp-S319 ett arf4*, *mp-12 ett arf5*, *fil ett arf4* and *hda19 ett arf4* inflorescences (**a,b,c** and **e**) as well as in *fil ett arf4* leaves (**d**). Relative expression of the *BP* class I KNOX gene in trimmed inflorescence apices or in fully expanded adult leaves was normalized over that of the *UBQ10* housekeeping gene. Shown are mean+/-SEM of three experiments.
f-j. Map of the *BP* locus and regions probed by ChIP qPCR **(f).** Green triangles FIL binding motif (AATNATAA)[3]; red triangles, ETT binding motif TGTCAT[4]. Chromatin immunoprecipitation (ChIP) to test ETT (**g**), MP (**h**), FIL (**i**) and HDA19 (**j**) binding to the *BP* locus. TA3 served as negative control. An MP-bound region of the *FIL* locus[5] served as positive control for MP ChIP (**h**), see also Fig. 4 c. ChIP was performed under identical conditions in the wild type (WT). Shown are mean+/-SEM of three experiments.
k. Anti-histone 3 lysine 27 acetylation (H3K27ac) relative to H3 levels at the *BP* locus in expanded leaves of wild type (WT), *fil/+ ett arf4* and *fil ett arf4*. *TA3* served as negative control. ChIP was performed under identical conditions in the wild type (WT). Shown are mean +/- SEM of three experiments.

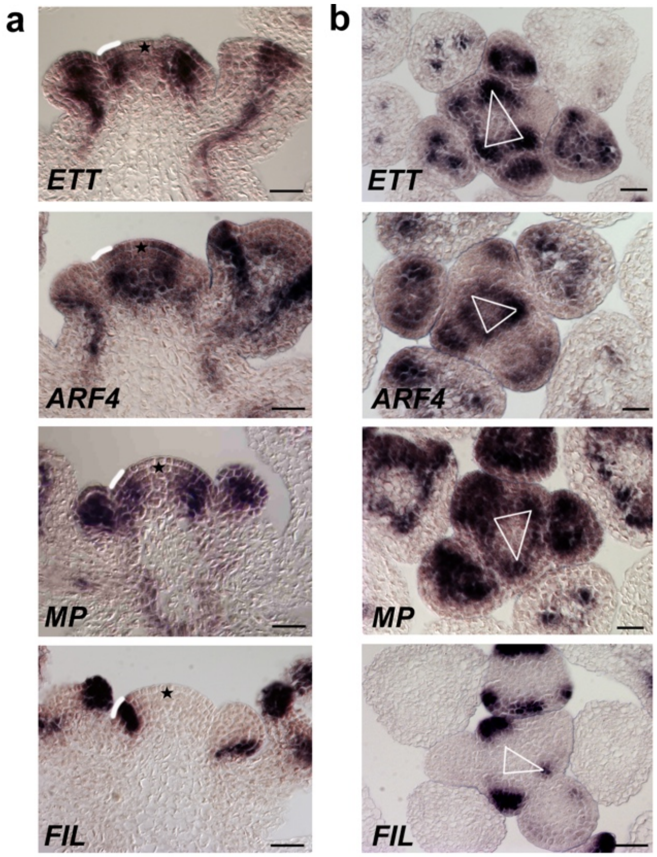

Supplementary Fig. 6. Expression of *ETT*, *ARF4*, *MP* and *FIL* in the reproductive shoot apices assayed by *in situ* hybridization.

a. Longitudinal sections through the reproductive shoot apex to monitor accumulation of *ETT*, *ARF4*, *MP* and *FIL* mRNA in the founder cells of reproductive primordia (white line). Asterisk – center of the shoot apical meristem (stem cells). Scale bar=30 µm

b. Transverse sections through the reproductive shoot apex to assess expression of *ETT*, *ARF4*, *MP* and *FIL* at the flanks of the shoot apex (white triangle). One of the triangle edges points to the founder cells of a reproductive primordium. Scale bar=30 µm

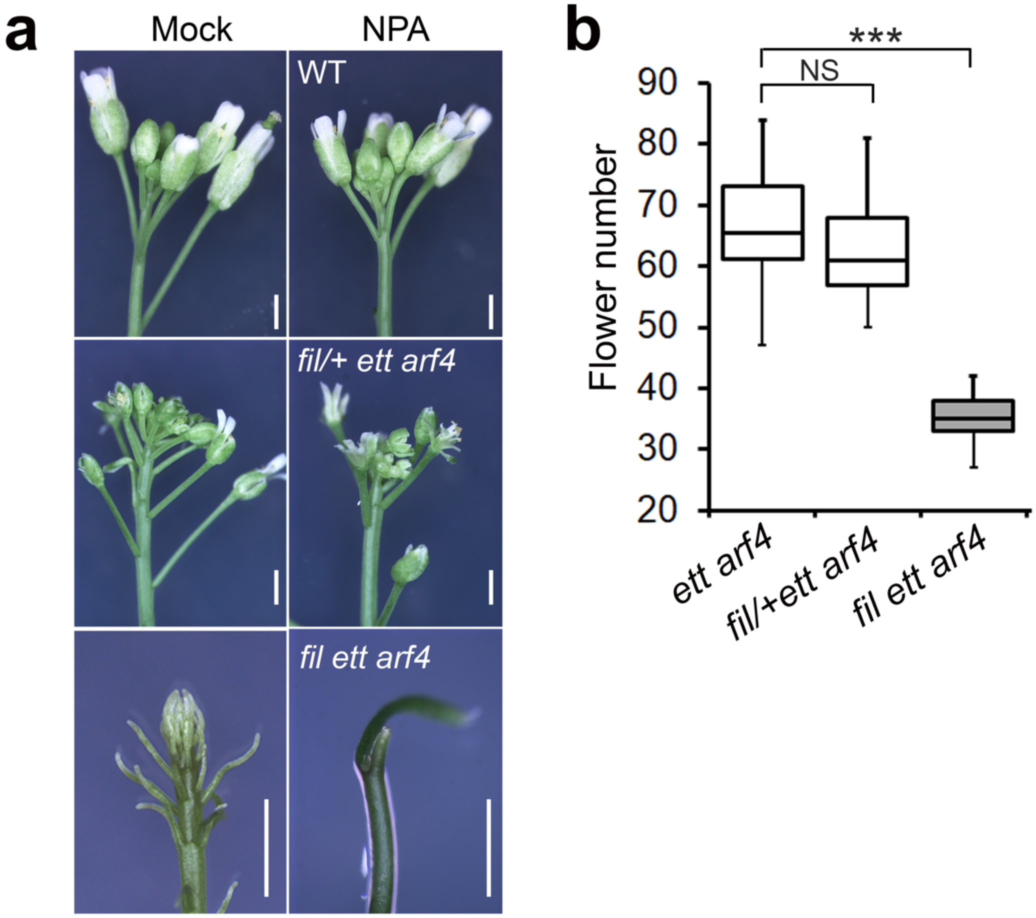

Supplementary Fig. 7 Inflorescence phenotype of *fil ett arf4*
a Phenotypes of *fil/+ ett arf4* and *fil ett arf4* inflorescences in the presence and absence of the auxin transport inhibitor NPA. NPA (500 nM), was applied from 12-days-of age until bolting. Scale bar = 1 mm. For quantification of the phenotype see Fig. 5b.
b Quantification of flower initiation in untreated *fil/+ ett arf4* and *fil ett arf4* compared to *ett arf4* plants. Box and whisker plot: Lower vertical bar= sample minimum. Lower box=lower quartile. Black line= median. Upper box= upper quartile. Upper vertical bar= sample maximum. NS=0.1867, *** P=0.00094*,* one-tailed Mann-Whitney U test. n=12 (*ett arf4*) n=11 (*fil/+ ett arf4*) n=5 (*fil ett arf4*).

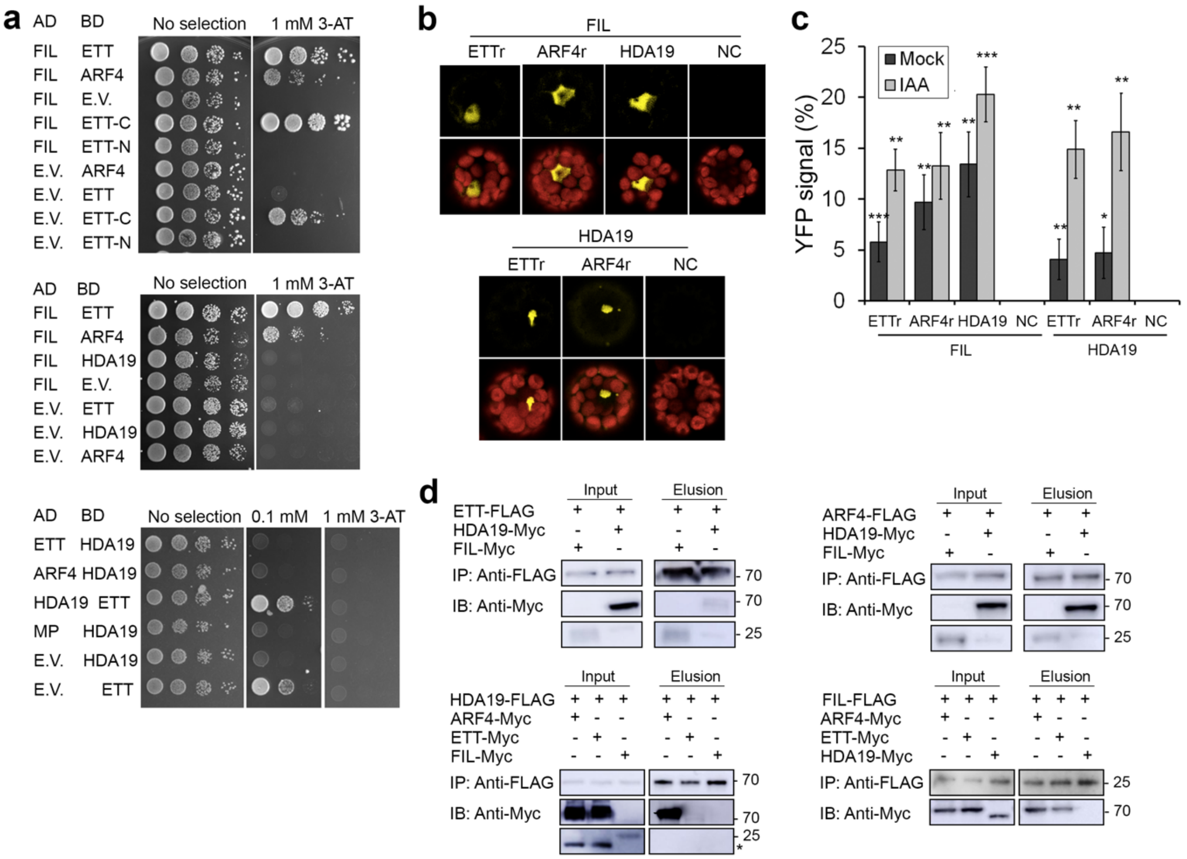

Supplementary Fig. 8 Physical interaction between FIL, ETT/ARF4 and HDA19

a Yeast-two hybrid assay to test for direct interaction between FIL and ETT/ARF4 and between HDA19 and FIL or ETT/ARF4. E.V. denotes empty vector (negative control). Yeast growth test (no selection, left) and interaction test (selective medium plus 0.1 mM or 1 mM 3-AT, right). FIL interacts with the C-terminal domain (aa 390-608) of ETT and with full-length ETT and ARF4. Neither FIL nor ETT or ARF4 interact with HDA19. This was expected as the interaction between the TFs and HDA19 is thought to be mediated by transcriptional co-repressor complexes[6-11].

b Confocal images of bifluorescence molecular complementation (BiFC) in leaf protoplasts confirms the interaction between FIL and ETT and FIL and ARF4. In addition, FIL, ETT and ARF4 associated with HDA19, presumably via co-repressor complexes present in the leaf protoplasts. Yellow signal: BiFC, red signal: chloroplast autofluorescence.

c Quantification of the interaction frequencies observed by BiFC as percent of protoplasts scored. Signal frequencies for interaction tests between FIL and ETTr, ARF4r or HDA19 are significantly increased relative to that between FIL and a negative control protein (SHORT-ROOT; AT4G37650). P=0.0016 and 0.00604 (FIL and ETTr, n=8 (no IAA) and n=5 (IAA)), P=0.005, 0.0054 (FIL and ARF4r, n=4 (no IAA), n=4 (IAA)), P=0.0052 and 0.0027 (FIL and HDA19, n=3 (no IAA) and n=6 (IAA)). Signal frequencies for interactions tests between HDA19 and ETTr or ARF4r are significantly increased relative to that between HDA19 and a negative control protein (SHORT-ROOT; AT4G37650). P=0.0054, 0.008 (ETTr and HDA19,

n=4 (no IAA) and n=3 (IAA)), P=0.014 and 0.0084 (ARF4r and HDA19, n=2 (no IAA) and n=3 (IAA)).

d. Analysis of protein-protein interaction by co-immunoprecipitation to test interactions between FIL and ETT or ARF4, and between HDA19 and ETT, ARF4 or FIL. Proteins transiently co-expressed in *N. benthaminana* leaves were immunoprecipitated using anti-FLAG beads. Western blot analysis was carried out using anti-FLAG or anti-Myc antibodies, respectively. The marks and numbers on the right of each blot indicate the molecular weight, while * indicates nonspecific bands. See Supplementary Fig. 12 for full size gel images.

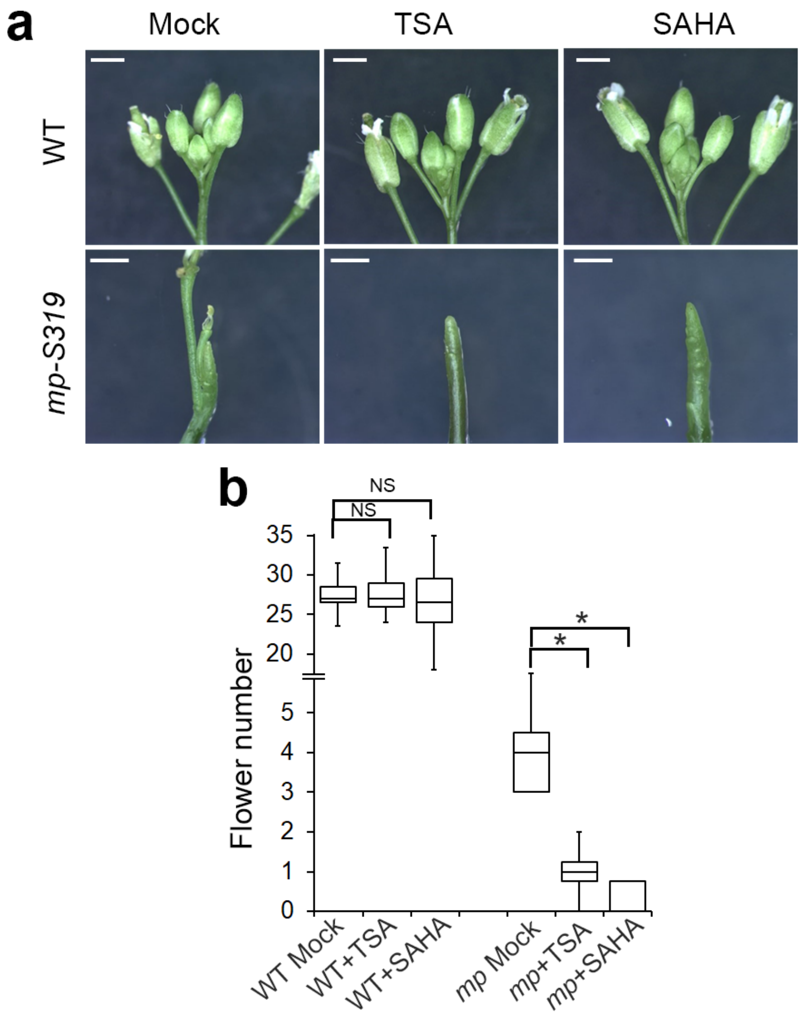

Supplementary Fig. 9 Histone deacetylase inhibitors enhance the flower initiation defect of *mp-S319*.

a Flower initiation phenotypes of wild type (WT) and the *mp-S319* hypomorph mutant treated with mock solution, with 5 µM Trichostatin A (TSA) or with 50 µM Suberoylanilide hydroxamic acid (SAHA) for 18 days. The phenotype was assayed after 20 days. Neither HDAC inhibitor treatment had an effect on the wild type. Scale bar = 1 mm.

b Quantification of the phenotypes shown in (**a**). Box and whisker plot: Lower vertical bar= sample minimum. Lower box=lower quartile. Black Line= median. Upper box= upper quartile. Upper vertical bar= sample maximum. NS=0.38 (WT mock vs TSA), NS=0.31(WT mock vs SAHA). ** P=0.0058 * P=0.01659 for *mp-S319* mock vs. TSA or vs. SAHA treatment, one-tailed Mann Whitney U test. n=11 (wild type mock), 9 (wild type TSA) and 10 (wild type SAHA). n=8 (*mp-S319* mock), 8 (*mp-S319* TSA) and 6 (*mp-S319* SAHA).

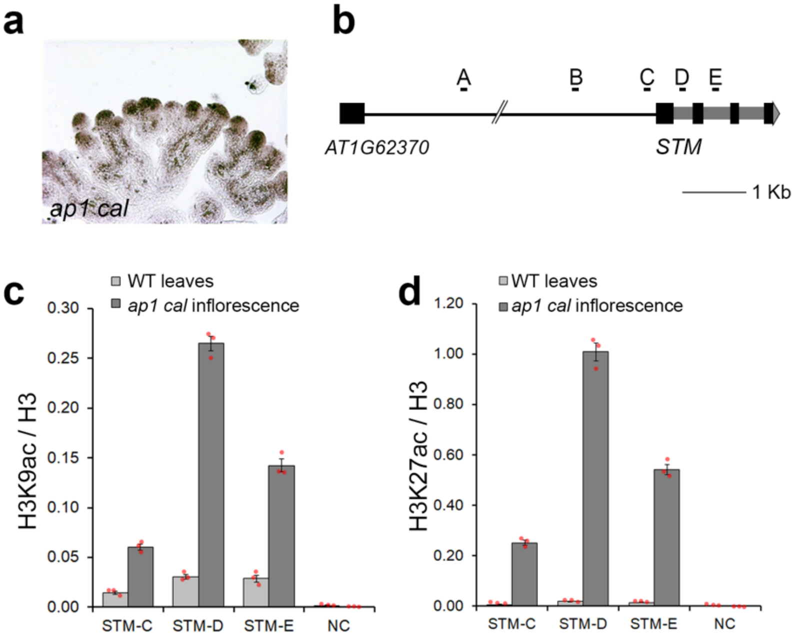

Supplementary Fig. 10 The active *STM* locus is marked with histone H3 lysine 9 and lysine 27 acetylation.

a *In situ* hybridization of *STM* in *ap1 cal*[12] inflorescences. *STM* is strongly expressed in the inflorescences and stage 2 flower meristems of the *ap1 cal* mutant shoot apex. *STM* is not expressed in expanded leaves[13].

b *STM* locus with conserved regions (A-E). See also Fig. 4.

c, d Presence of acetylated histone H3 lysine 9 (**c**) and acetylated histone H3 lysine 27 (**d**) relative to that of histone H3 at the *STM* locus. Expanded wild-type Columbia leaves and *ap1 cal* L*er* inflorescences were sampled. Both lysines are strongly acetylated *in ap1 cal* inflorescences, where *STM* is highly expressed, with overall higher levels of H3K27ac. Shown are mean+/-SEM of one representative of three experiments.

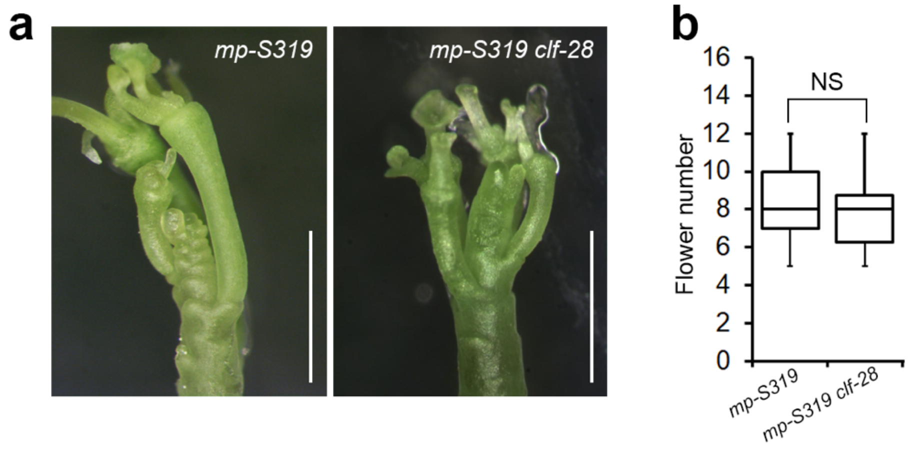

Supplementary Fig. 11 Inflorescence phenotype of *mp-S319 clf-28*

a Flower initiation phenotypes of *mp-S319* compared to *mp-S319* also carrying a loss-of-function allele for the PRC2 complex component *CLF*, *clf-28*[14]. Scale bar = 1 mm

b Quantification of the flower initiation in *mp-S319 clf-28* compared to *mp.* NS P=0.3015, one-tailed Mann–Whitney U test. n=17 and 14 for *mp-S319* and *mp-S319 clf-28*.

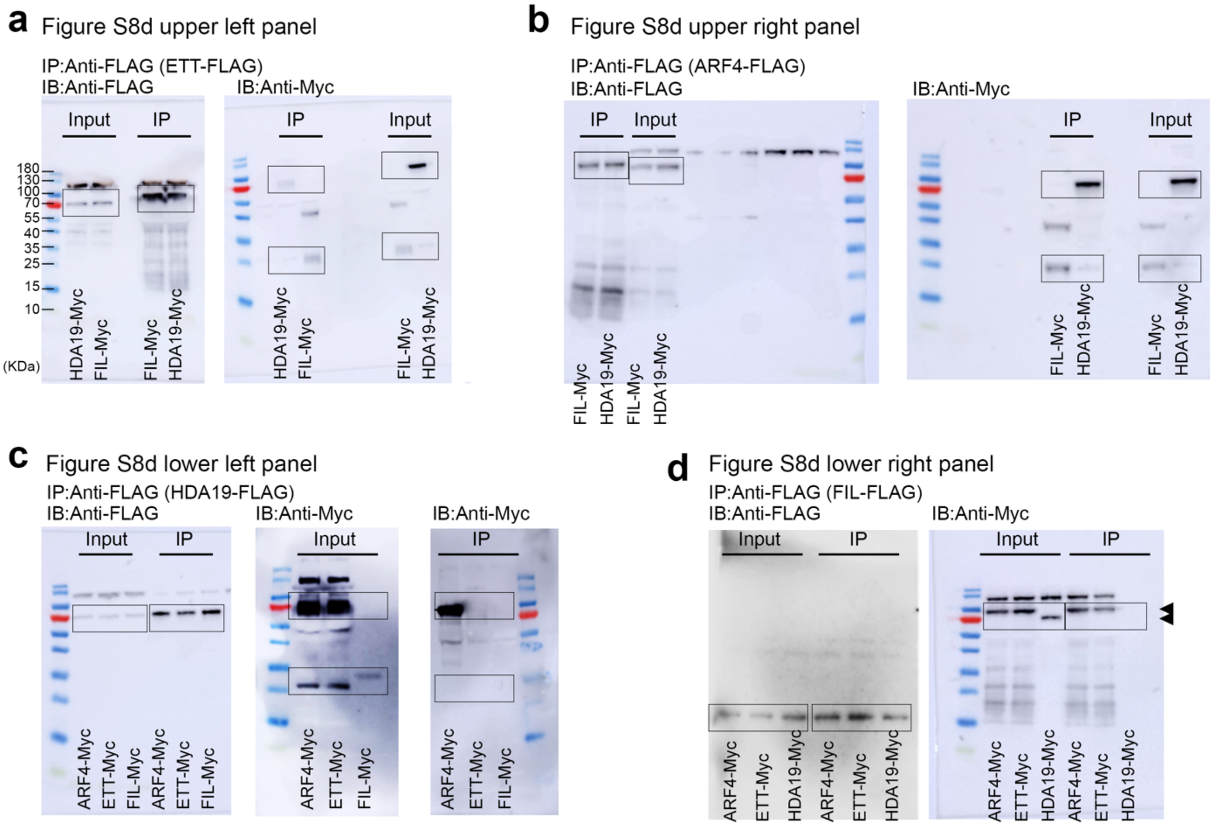

Supplementary Fig. 12 Full size gel blot images for co-immunoprecipitation experiments
a-d The black frames surround areas that were cropped to assemble the panels in Supplementary Figure 8d. The marker lane on the left of each blot and the numbers in panel a indicate the molecular weight of the protein ladder.

Supplementary Tables

Supplementary Table 1. List of primers used in this study

Primer Name	Sequence 5’ to 3’	
**qRT-PCR**		
STM-RT-F	CCCTTGCTCCTCTTCCTCTT	
STM-RT-F	CTCTAGCCTCGCCACAACCT	
IPT7-RT-F	TCGAAACTCTCAGCGAACAA	
IPT7-RT-R	TCGACCCAAATGAAACAACA	
BP-RT-F	CCTTATCGTCTCGGTCCATAAG	
BP-RT-R	GTTGTAACAAGAAAGCAACGAGAGG	
FIL-RT-F	ACCATCTTTCATGGATCTTCA	
FIL-RT-F	AATCGGTTATATGCGGATGG	
UBQ10-RT-F	ATGGGTCCTTCAGAGAGTCCT	
UBQ10-RT-R	CTTGGTCCTAAAGGCCACCT	
**Constructs for generating transgenic plants, yeast two- hybrid and BiFC**		
ETT-F	caccATGGGTGGTTTAATCGATCTG	
ETT-R	CTAGAGAGCAATGTCTAGCAAC	
ARF4-F	caccATGGAATTTGACTTGAATACTG	
ARF4-R	TCAAACCCTAGTGATTGTAGGAG	
FIL-F	caccATGTCTATGTCGTCTATGTCC	
FIL-R	TTAATAAGGAGTCACACCAACG	
HDA19-F	caccATGGATACTGGCGGCAATTC	
HDA19-R	TTATGTTTTAGGAGGAAACGCCTG	
FIL promoter-F	gtggtctcaGGAGgtatcacatcacgtttgagttacg	
FIL gene-R no STOP	gtggtctcTcgaaacATAAGGAGTCACACCAACGTTAGCAGC	
**ChIP assays**		
STM-A-F	CAAATCTTTCAATAGTAGACAGAGG	
STM-A-R	GAATAAAGGAGGAAAGGTGAGGT	

STM-B-F	GATGGGTTCCTTGTAATCTCTCTC	
STM-B-R	TGCCTTCACTGCCATTTCTG	
STM-C-F	CAATGTGGAAAAGGAAGCTGATTG	
STM-C-R	CACTTTGGCTTTGCTATATAGC	
STM-D-F	GAAGAGAAAGATTCTAGAAACCC	
STM-D-R	GTCCTAATCTTCAAGCCTACATAC	
STM-E-F	TGGTTGTTACTAGGGTTAGTG	
STM-E-R	CCAAAACAAACGGAGAAGAAAG	
FILupstream-F	GGGTTTGTATACCCCGATCC	
FILupstream-R	CCATTGAACCATCACCGATA	
BP-A-F	CTTTTGTAGCGCGCATCTCTCG	
BP-A-R	GTTTGGTTTAGTGTTTCACTTGTTGAC	
BP-B-F	CAGAAGAGAGAAGCCTTTGCC	
BP-B-R	GACGTCGTTTGCTTTGGGAGC	
BP-C-F	CCATGTTAATGAGAGCCATCC	
BP-C-R	CTATCAACCACATCAGGTGGAG	
TA3-F	CTGCGTGGAAGTCTGTCAAA	
TA3-R	CTATGCCACAGGGCAGTTTT	

Supplementary References

1 Yanai, O. *et al.* Arabidopsis KNOXI proteins activate cytokinin biosynthesis. *Curr Biol* **15**, 1566-1571 (2005).

2 Jasinski, S. *et al.* KNOX action in Arabidopsis is mediated by coordinate regulation of cytokinin and gibberellin activities. *Curr Biol* **15**, 1560-1565 (2005).

3 Franco-Zorrilla, J. M. *et al.* DNA-binding specificities of plant transcription factors and their potential to define target genes. *Proc Natl Acad Sci U S A* **111**, 2367-2372 (2014).

4 Simonini, S. *et al.* A noncanonical auxin-sensing mechanism is required for organ morphogenesis in Arabidopsis. *Genes Dev* **30**, 2286-2296 (2016).

5 Wu, M. F. *et al.* Auxin-regulated chromatin switch directs acquisition of flower primordium founder fate. *eLife* **4**, e09269 (2015).

6 Pfluger, J. & Zambryski, P. The role of SEUSS in auxin response and floral organ patterning. *Development* **131**, 4697-4707 (2004).

7 Causier, B., Ashworth, M., Guo, W. & Davies, B. The TOPLESS interactome: a framework for gene repression in Arabidopsis. *Plant Physiol* **158**, 423-438 (2012).

8 Gonzalez, D., Bowen, A. J., Carroll, T. S. & Conlan, R. S. The transcription corepressor LEUNIG interacts with the histone deacetylase HDA19 and mediator components MED14 (SWP) and CDK8 (HEN3) to repress transcription. *Molecular and cellular biology* **27**, 5306-5315 (2007).

9 Stahle, M. I., Kuehlich, J., Staron, L., von Arnim, A. G. & Golz, J. F. YABBYs and the transcriptional corepressors LEUNIG and LEUNIG_HOMOLOG maintain leaf polarity and meristem activity in Arabidopsis. *Plant Cell* **21**, 3105-3118 (2009).

10 Krogan, N. T., Hogan, K. & Long, J. A. APETALA2 negatively regulates multiple floral organ identity genes in Arabidopsis by recruiting the co-repressor TOPLESS and the histone deacetylase HDA19. *Development* **139**, 4180-4190 (2012).

11 Sridhar, V. V., Surendrarao, A., Gonzalez, D., Conlan, R. S. & Liu, Z. Transcriptional repression of target genes by LEUNIG and SEUSS, two interacting regulatory proteins for Arabidopsis flower development. *Proc Natl Acad Sci U S A* **101**, 11494-11499 (2004).

12 Kempin, S. A., Savidge, B. & Yanofsky, M. F. Molecular basis of the cauliflower phenotype in Arabidopsis. *Science* **267**, 522-525 (1995).

13 Kumaran, M. K., Bowman, J. L. & Sundaresan, V. YABBY polarity genes mediate the repression of KNOX homeobox genes in Arabidopsis. *Plant Cell* **14**, 2761-2770 (2002).

14 Doyle, M. R. & Amasino, R. M. A single amino acid change in the enhancer of zeste ortholog CURLY LEAF results in vernalization-independent, rapid flowering in Arabidopsis. *Plant Physiol* **151**, 1688-1697 (2009).
